# TGF-β1-Mediated PD-L1 Glycosylation Contributes to Immune Escape *via* c-Jun/STT3A Pathway in Nasopharyngeal Carcinoma

**DOI:** 10.3389/fonc.2022.815437

**Published:** 2022-03-04

**Authors:** Xue-Min Ma, Yun-Fan Luo, Fang-Fang Zeng, Chang Su, Xiong Liu, Xiang-Ping Li, Juan Lu

**Affiliations:** ^1^ Department of Otolaryngology-Head and Neck Surgery, Nanfang Hospital, Southern Medical University, Guangzhou, China; ^2^ Department of Otolaryngology-Head and Neck Surgery, Shenzhen Second People’s Hospital, The First Affiliated Hospital of Shenzhen University, Shenzhen, China

**Keywords:** TGF-β1, PD-L1, glycosylation, nasopharyngeal carcinoma, immunotherapy

## Abstract

Immunotherapy targeting programmed death ligand-1/programmed cell death protein-1 (PD-L1/PD-1) has achieved great success in multiple cancers, but only a small subset of patients showed clinical responses. Recent evidences have shown that post-translational modification of PD-L1 protein could regulate its protein stability and interaction with cognate receptor PD-1, thereby affecting anticancer immunotherapy in several solid tumors. However, the molecular mechanisms underlying how PD-1/PD-L1 expression is regulated still remain unclear in nasopharyngeal carcinoma (NPC). Here, we found N-glycosylation of PD-L1 in NPC cells and tissues. Mechanistically, we showed that STT3A transferred N-linked glycans to PD-L1, and TGF-β1 could positively regulate STT3A expression through activating c-Jun to bind to STT3A promoter. Functional assays showed that inhibition of TGF-β1 resulted in a decrease of glycosylated PD-L1 and enhanced cytotoxic T-cell function against NPC cells. Analysis of clinical specimens revealed that the expression of STT3A was positively correlated with TGF-β1 and c-Jun, and high STT3A expression was positively correlated with a more advanced clinical stage. Altogether, TGF-β1 activated c-Jun/STT3A signaling pathway to promote N-glycosylation of PD-L1, thus further facilitating immune evasion and reducing the efficacy of cancer immunotherapy. As such, all these data suggested that targeting TGF-β1 pathway might be a promising approach to enhance immune checkpoint blockade, and simultaneous blockade of PD-L1 and TGF-β1 pathways might elicit potent and superior antitumor activity relative to monotherapies.

## Introduction

Nasopharyngeal carcinoma (NPC) is a kind of epithelial squamous cell carcinoma that originates from the nasopharyngeal mucosa with a high incidence in South China and Southeast Asia. NPC is characterized by prevailing Epstein-Barr virus (EBV) infection and a heavy infiltration of immune cells in tumor microenvironment (1). At present, metastasis and recurrence are the main cause of death in NPC (1). Immunotherapy involving blocking programmed death ligand-1/programmed cell death protein-1 (PD-L1/PD-1) immune checkpoints has achieved great success in many solid tumors (2–6). Due to the efficacy of improving immunosuppressive microenvironment, anti-PD-1/PD-L1 treatment is suggested to be a promising therapeutic method for recurrent or metastatic NPC patients. Although PD-L1 is extremely highly expressed in NPC tissues (7), the response rate of PD-L1/PD-1 blocking therapy in NPC is only 20-30% (8–10). Moreover, an inconsistency occurs between the expression level of PD-L1 and the therapeutic effect (11). Therefore, a better understanding of the molecular mechanisms underlying how PD-1/PD-L1 expression is regulated will provide new insights to improve anti-PD-1/PD-L1 efficacy in NPC.

PD-L1, also known as CD274 or B7-H1, is a transmembrane glycoprotein expressed by tumor cells, macrophages and T cells. PD-L1 inhibits T cells activity and promotes immune evasion through binding to its receptor PD-1 on T cells (12). Recent studies have found that post-translational modifications (PTMs) of PD-L1 have played significant roles in modulating immunosuppression in cancers. PTMs of PD-L1, such as phosphorylation, N-glycosylation, acetylation and poly-ubiquitination, are known to emerge as important mechanisms in regulating its stability, translocation, and interactions with other proteins and directly affect PD-L1-mediated immune tolerance (13–15). A recent report indicated that glycosylation of PD-L1 led to the block of recognition of PD-L1/PD-1 by monoclonal antibodies and a low response rate of anti-PD-L1/PD-1 therapy in breast cancer (11).

TGF-β is a multifunctional cytokine with an important role in both physiologic and pathologic processes, including cancer. The aberrantly upregulated production of TGF-β has been strongly implicated in tumor progression, angiogenesis, and metastasis, as well as immune escape. TGF-β has been regarded as a critical immunosuppressive cytokine, which suppresses the antitumor activity of effector cells, including CD8+ T cells, natural killer (NK) cells, and macrophages (16). Several studies have indicated that TGF-β-mediated inhibitory immune microenvironment weakens the ability of PD-L1/PD-1 inhibitors to rebuild anti-tumor immune response, and ultimately leads to the resistance to PD-1/PD-L1 inhibitors (17–19). A recent report found that epithelial-mesenchymal transition (EMT) driven by TGF-β caused PD-L1 glycosylation by activating β-catenin to transcriptionally upregulate the expression of N-glycosyltransferase STT3, and eventually promoted immune escape in cancer stem cells (CSCs) (20). Furthermore, our previous study has revealed that enrichment of TGF-β1 in NPC microenvironment promotes immune escape by inducing chemotactic migration of Regulatory T (Treg) cells to remodel the inhibitory immune microenvironment *via* the TGF-β1-SMAD3-PI3K-AKT-c-JUN-miR-200a-CXCL12-CXCR4 axis (21, 22). Based on these previous results, we wonder whether TGF-β1 exerts synergistic effects on the PD-L1 induction and attenuates tumor response to PD-L1 blockade in NPC.

In this study, we verified the N-glycosylation of PD-L1 in NPC and further investigated whether TGF-β1 induced glycosylation of PD-L1 to promote immune escape, which may contribute to develop more effective immunotherapy strategies and improve the survival rate of NPC patients.

## Methods and Materials

### Cell Culture

All the NPC cell lines including 5-8F, CNE1, CNE2, SUNE1, HONE1, and HNE1 were obtained from the Cancer Research Center of Southern Medical University. Cells were cultured in RPMI 1640 medium (11875176, Gibco, USA) with supplement of 10% fetal bovine serum (2053264, BI, Israel), 100 mg/ml streptomycin and 100 U/ml penicillin(C125C5, NCM Biotech, China) in an incubator at 5% CO2, 37°C. The PD-L1-knockdowned cells and c-Jun-overexpressed cells were cultured with antibiotic [puromycin (1μg/ml) (P8230, Solarbio, China)] while the Flag-PD-L1 cells and c-Jun- and STT3A-knockdowned cells were cultured with neomycin (400μg/ml) (G8160, Solarbio, China).

### Western Blotting

The radio immunoprecipitation assay lysis buffer (RIPA) (P0013B, Beyotime, China) containing a protease-inhibitor cocktail (HY-K0010, MCE, USA) were used to extract proteins from cells. The proteins were lysed in SDS-loading buffer (FD006, Fdbio, China), then 20μg proteins were resolved on a 10% sodium dodecyl sulphate–polyacrylamide gel electrophoresis and transferred to polyvinylidene fluoride (PVDF) membrane (IPVH00010, Millipore, USA). The membrane was incubated with primary antibodies against PD-L1(13684, Cell Signaling Technology, USA), FLAG (80010-1-RR, Proteintech, USA), STT3A (12034-1-AP, Proteintech, USA), c-Jun (AF6089, Affinity, USA), HA (51064-2-AP, Proteintech, USA) and GAPDH (60004-1-Ig, Proteintech, USA) at a dilution of 1:1000, followed by incubation with species-specific (rabbit or mouse) HRP-conjugated secondary antibodies at a dilution of 1:5000. The immunoreactive bands were visualized by Omni ECL reagent (SQ101, EpiZyme, China).

### Protein Deglycosylation

The protein lysate exacted from samples using RIPA was undergo glycoprotein denaturation at 100°C for 10minwith 1μl 10X Glycoprotein Denaturing Buffer and ddH_2_O, and then incubated with PNGase F (P0704, NEB, USA) or O-glycosidase (P0733, NEB, USA) for 16h with 2μl 10X GlycoBuffer, 2μl 10% NP-40 and 5ul ddH_2_O or 2μl neuraminidase and 3ul ddH_2_O. The extent of deglycosylation was assessed by mobility shifts on SDS-PAGE gels.

### Generation of Stable Cells Using Lentiviral Infection

The lentiviral –based short-hairpin RNA (shRNA) (Genechem, China) was used to knock down PD-L1, c-Jun and STT3A of 5-8F and CNE1 cells. Based on the knockdown of endogenous PD-L1, a pCMV-Flag-PD-L1 overexpressing plasmid was generated to reconstitute Flag-PD-L1. A pCMV-HA-c-Jun overexpressing plasmid was used to culture stable overexpressing c-Jun cell lines.

### RNA Extraction, Reverse Transcription and qRT-PCR

Total RNA of cells was extracted RNA isolation Kit (RC112-01, Vazyme, China) and reversely transcribed to cDNA using a reverse transcription kit (R323-01, Vazyme, China). ChamQ SYBR RT-qPCR Master Mix (Low ROX Premixed) (Q331-02, Vazyme, China) was used to used make a 20μL reaction amplification system to perform reverse transcription quantitative polymerase chain reaction (RT-qPCR) on an ABI QuantStudio6 System. All samples were normalized to the internal control GAPDH mRNA, and the relative expression levels were calculated based on the 2^−△△CT^ method. The primer sequences for RT-qPCR are shown in **Supplementary Table 2**.

### ConA Lectin Binding Assay

Immunoprecipitated PD-L1 proteins were subjected to SDS-PAGE gels, transferred onto PVDF (IPVH00010, Millipore, USA), blocked with RIPA buffer for 1h at room temperature, and then incubate by peroxidase-conjugated ConA lectin (dilute to 0.1 μg/ml with RIPA buffer) (L6397, Sigma-Aldrich, USA) for 2h. Wash the membrane with RIPA buffer and detection using a chemiluminescent substrate.

### Dual-Luciferase Assay

For the preparation of the 293T cells for transfection, they (1 × 105 cells) were grown by incubation on a 24-well plate for 24 hours. The control pcDNA3.1 plasmid (4.0 μg/ml) or the c-Jun plasmid (4.0 μg/ml), together with the control vector, pGL3-basic (4.0 μg/ml) or the STT3A plasmid (4.0 μg ml) were added into the medium with Lipofectamine 2000 (116680119, Invitrogen, USA). Cell lysates were collected after 36h incubation and firefly/renilla luciferase values were measured by the Dual-Luciferase Reporter Assay System (E1910, Promega, USA).

### CHIP-PCR

The EZ-Magna ChIP™ A/G (17-10086, Millipore, USA) was used to perform CHIP experiment according to the manufacturer’s instruction. Anti-c-Jun antibodies (AB40766, Abcam, USA) were used for immunoprecipitation and two primer sets (**Supplementary Table 3**) designed against human STT3A promoter were used to detect the bound DNA.

### Co-Culture Experiment

Jurkat T cells were activated by Dynabeads Human T-Activator CD3/CD28(11161D, Gibco, USA), and were then co-cultured with 5-8F or CNE1 cells pretreated with SB431542 (20μM) (HY-431542, MCE, USA) or tunicamycin (15μg/ml) (T8480, Solarbio, China) for 48h at 3:1 (Jurkat: 5-8F/CNE1) ratio for 6h. The death rate of tumor cells was detected by CFSE/PI Double Stain Kit (BB4214, Bestbio, China). The medium supernatant of the co-culture system was collected for detecting the level of IL-2 by the ELISA kit (70-EK102, MultiSciences, China) according to manufacturer’s instructions.

### Clinical Samples and Immunohistochemistry

The NPC tissue specimens (n=36) were collected from the Nanfang Hospital of Southern Medical University (Guangzhou, China). These tissue specimens were from biopsy samples of NPC patients (pathologically confirmed) in 2021. The TNM classification was performed according to the 8th edition of the UICC/AJCC staging criteria. The clinical characteristics of these 36 patients with NPC are shown in **Supplementary Table 3**. This study was approved by the Ethics Committee of Nanfang Hospital of Southern Medical University. Formalin-fixed, and paraffin-embedded tissues were sectioned at 4 mm thickness, then harvested and fixed in 4% paraformaldehyde overnight at 4°C. The antigen blocking was performed using 10% goat serum (SP-9000, Zsbio, China). The sections were probed overnight at 4°C with primary anti-TGF-β1 (PA5-40772, Invitrogen, USA), anti-c-Jun (AF6089, Affinity, USA) and anti-STT3A (sc-100796, Santa Cruz, USA) antibodies. DAB color reagent kit (ZLI-9018, Zsbio, China) was used for staining. The numbers of positive cells were measured by two independent pathologists through five randomly selected fields at ×400 magnification.

### Statistical Analysis

The SPSS 20.0 software was used for statistical analyses. All data were from at least three independent experiments. The comparison between two independent groups of data following a normal distribution is analyzed by two-tailed student’s t-test. The relationship between STT3A and TGF-β1 or c-Jun was analyzed by the Spearman’s correlation analysis. The data are shown as the mean ± SEM unless otherwise. *p*-values of <0.05 were considered statistically significant.

## Results

### PD-L1 Was N-glycosylated in Nasopharyngeal Carcinoma

**Figure 1:**
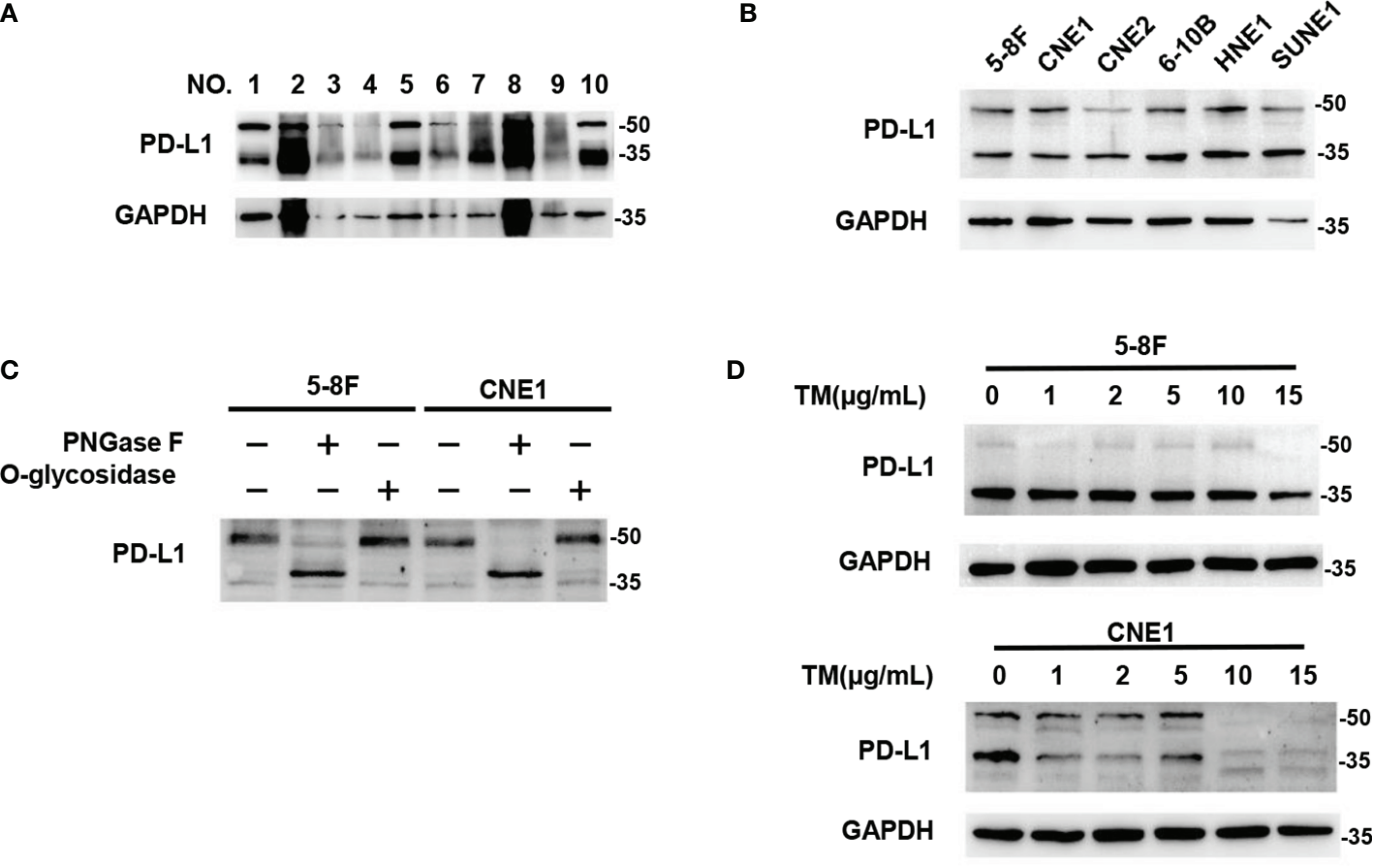
PD-L1 was N-glycosylated in Nasopharyngeal Carcinoma. **(A)** Western blot analysis of PD-L1 in NPC patient samples. **(B)** Western blot analysis of PD-L1 in 6 NPC cells including 5-8F, CNE1, CNE2, 6-10B, HNE1, SUNE1. **(C)** Glycosylation pattern of PD-L1 protein in 5-8F and CNE1 cells. Cell lysates were treated with PNGase F or O-glycosidase and analyzed by western blot analysis. **(D)** Western blot analysis of PD-L1 in 5-8F and CNE1 cells treated by Tunicamycin (TM) with increasing concentrations.

We analyzed PD‐L1 expression in human NPC tissues and six different NPC cell lines by western blot, and found that most of PD-L1 expression was presented as two kinds of molecular weight size, which were distributed at around 50kDa and ~ 40kDa respectively (**Figures 1A, B**). These findings were consistent with previous results in breast cancer (23), suggesting that the 50-kDa form in NPC might be glycosylated PD-L1, and the ~40-kDa form might be non-glycosylated PD-L1. To further confirm the glycosylation of PD-L1 in NPC, we treated 5-8F and CNE1 cells with recombinant glycosidase (peptide-N-glycosidase F; PNGase F) or O-glycosidase. Compared with O-glycosidase-treated groups, virtually all PD-L1 migrated from 50 kDa to around 40 kDa upon PNGase F treatment in 5-8F and CNE1 cells (**Figure 1C**). The 50-kDa PD-L1 in 5-8F and CNE1 cells was also restrained by the N-glycosylation inhibitor tunicamycin (TM) (**Figure 1D**). Taken together, the results suggested that PD-L1 in NPC was N-glycosylated, and 50-kDa PD-L1 was glycosylated form and ~40-kDa PD-L1 was un-glycosylated form.

### Inhibition of TGF-β1 by SB431542 Reduced PD-L1 Glycosylation *In Vitro*

**Figure 2:**
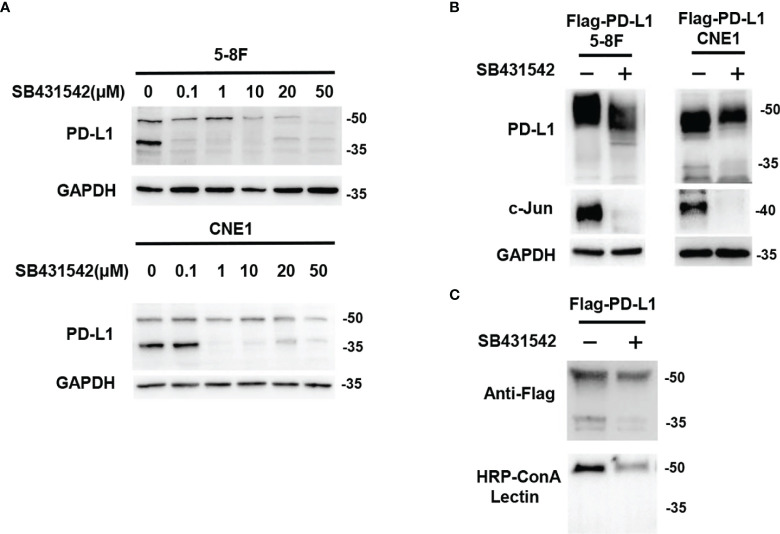
Inhibition of TGF-β1 by SB431542 Reduced PD-L1 Glycosylation *in Vitro*. **(A)** Western blot analysis of PD-L1 in 5-8F and CNE1 cells treated by SB431542 with increasing concentrations. **(B)** Western blot analysis of PD-L1 and c-Jun in exogenous Flag-PD-L1 expressing 5-8F and CNE1 cells with the treatment of 20μM SB431542. **(C)** The glycosylation status of PD-L1 protein purified from SB431542 treating cells was analyzed by ConA lectin binding assay.

To explore the relationship between TGF-β1 and PD-L1 glycosylation, SB431542, a TGF-β type I receptor inhibitor, was used to block the effect of TGF-β1 in 5-8F and CNE1 cells. The results showed that inhibition of TGF-β1 by SB431542 reduced PD-L1 expression (**Figure 2A**). Then, 5-8F and CNE1 cells stably overexpressing exogenous Flag-tagged-PD-L1 were constructed, in which the expression of PD-L1 was independently of the transcriptional regulation of endogenous PD-L1. If PD-L1 protein expression was upregulated or downregulated by any cytokines in those cells, it likely occurred through post-translational modifications. The results showed that the exogenous PD-L1 in Flag-PD-L1 5-8F and CNE1 cells could be reduced by SB431542 (**Figure 2B**). Moreover, PD-L1 lost its binding ability to ConA lectin after the treatment of SB431542 (**Figure 2C**). In summary, these results demonstrated that blocking the effect of TGF-β1 by SB431542 suppressed PD-L1 glycosylation *in vitro*.

### SB431542 Downregulated Glycosylation of PD-L1 *via* C-Jun/STT3A Pathway *In Vitro*

**Figure 3:**
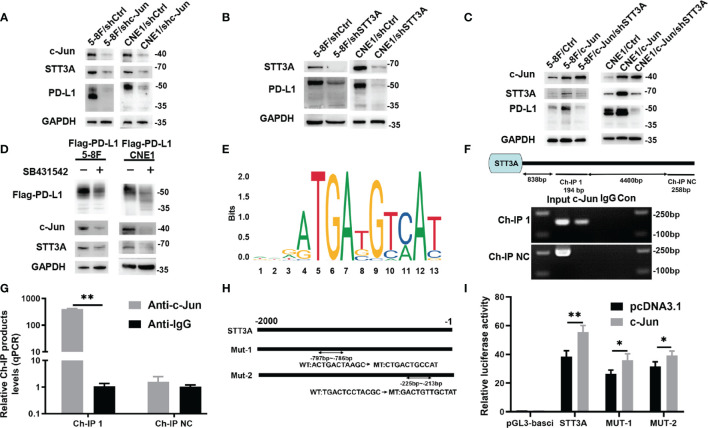
SB431542 Downregulated Glycosylation of PD-L1 *via* C-Jun/STT3A Pathway *in Vitro* and C-Jun was a Direct Transcriptional Regulator of STT3A. **(A)** Western blot analysis of c-Jun, STT3A and PD-L1 after silencing c-Jun in 5-8F and CNE1 cells. **(B)** Western blot analysis of STT3A and PD-L1 after silencing STT3A in 5-8F and CNE1 cells. **(C)** Western blot analysis of c-Jun, STT3A and PD-L1 after overexpressing c-Jun with(out) silencing STT3A in 5-8F and CNE1 cells. **(D)** Western blot analysis of PD-L1, c-Jun and STT3A in exogenous Flag-PD-L1 expressing 5-8F and CNE1 cells with the treatment of 20μM SB431542. **(E)** The binding motif of c-Jun from JASPAR database. **(F)** The CHIP-PCR assay was used to assess the binding of STT3A promoter region. **(G)** Anti-c-Jun-pulled down chromatins were analyzed by qRT-PCR. **(H)** A diagram showing the relationship of full-length and mutant STT3A promoters. **(I)** Dual-luciferase reporter gene assay was performed to indicate the interaction between c-Jun and STT3A. Bars, mean ± SD, *p<0.5, **p<0.01.

Although SB431542 reduced PD-L1 glycosylation, the exact mechanism was still unclear. We verified that SB431542 also downregulated c-Jun expression in Flag-PD-L1 5-8F and CNE1 cells (**Figure 2B**). In our previous study, we also found that EBV-EBNA1 promoted the chemoattraction of Treg cells by governing the protein-protein interactions of the SMAD3/c-JUN complex in a TGF-β1-dependent manner in NPC (21). Furthermore, we detected the expression of 15 glycosyltransferases related to PD-L1 glycosylation in 5-8F cells after the treatment of TGF-β1, and found that STT3A had the most significant increase in transcription levels (**Figure S1**). We assumed that SB431542 could downregulate PD-L1 glycosylation by blocking TGF-β1 receptor to inhibit c-Jun and STT3A. In order to explore the exact mechanism, we knocked down c-Jun expression in 5-8F and CNE1 cells with shRNA, and the results showed that silencing c-Jun inhibited STT3A expression and PD-L1 glycosylation (**Figure 3A**). Next, after knockdown of STT3A, glycosylated PD-L1 in 5-8F and CNE1 cells were also downregulated (**Figure 3B**). Furthermore, we overexpressed c-Jun and then knocked down STT3A in 5-8F and CNE1 cells. The data demonstrated that upregulation of PD-L1 glycosylation caused by c-Jun overexpression was reversed by knockdown of STT3A (**Figure 3C**). Altogether, these results demonstrated that SB431542 reduced PD-L1 glycosylation through c-Jun/STT3A pathway, and c-Jun was an upstream regulator of STT3A (**Figure 3D**).

### C-Jun Was a Direct Transcriptional Regulator of STT3A

C-Jun is a major component of the dimeric transcription factor activator protein-1 (AP-1) (24), which regulates the expression of target genes at transcriptional level by binding to their promoters. Therefore, we speculated that c-Jun might regulate the expression of STT3A at the transcription level. We used UCSC and JASPAR databases to predict c-Jun-binding sites in the STT3A promoter (**Figure 3E**). A chromatin immunoprecipitation (CHIP) assay was performed to validate the interaction between c-Jun and STT3A promoter. The data of CHIP-PCR showed that the predicted STT3A binding sequence was successfully pulled down by anti-c-Jun group (**Figure 3F**). And RT-qPCR analysis of the pulled down chromatins demonstrated that c-Jun could bind to STT3A promoter through the putative binding site in the anti-c-Jun group but not in the IgG group (**Figure 3G**). Then, we constructed a series of luciferase reporter vectors containing the full-length of the wild type (WT) or mutant (Mut) c-Jun promoter. The mutant STT3A promoters contained two degenerate c-Jun-binding elements, including Mut-1 (CTGACTGCCAT) and Mut-2 (GACTGTTGCTAT) (**Figure 3H**). Luciferase reporter assays displayed that c-Jun promoted the luciferase activity of STT3A promoter but not the control vector. Furthermore, Mut-1 and Mut-2 reduced the STT3A transactivation, indicating that Mut-1 and Mut-2 were the effective binding sites (**Figure 3I**). The above results revealed that c-Jun regulated the expression of STT3A at transcriptional level by binding to its promoter.

### STT3A Expression Was Positively Correlated With TGF-β1 and C-Jun in NPC Tissues, and High STT3A Expression Was Associated With a Advanced Stage in NPC

**Figure 4:**
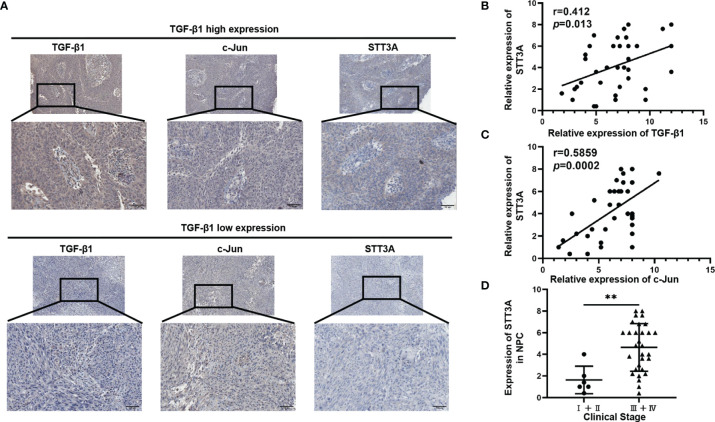
STT3A Expression was Positively Correlated with TGF-β1 and C-Jun in NPC Tissues, and High STT3A Expression was Associated with a more advanced stage in NPC. **(A)** Representative pictures of c-Jun and STT3A expression in NPC patients in TGF-β1-low and high expression groups. Scale bars represented 100μm. **(B, C)** Pearson correlation analysis of the association between TGF-β1 and STT3A (r=0.412; *p*=0.013) and between c-Jun and STT3A (r=0.5859; *p*=0.0002) in NPC tissues. **(D)** Comparison of STT3A levels in 36 NPC patients with different clinical stages. **p<0.01.

To further investigate the relationship among TGF-β1, c-Jun and STT3A in human NPC tissues, we analyzed the correlations among them in NPC tissues. As expected, STT3A expression was respectively positively correlated with TGF-β1 (r=0.412, p=0.013) and c-Jun (r=0.5859, *p*=0.0002) expression (**Figures 4A–C**). Meanwhile, the NPC patients with early-stage tumors (clinical stage I and II) had significantly downregulated expression of STT3A compared to those with a more advanced stage (clinical stage III and IV) (*p*<0.01) (**Figure 4D**), indicating that high STT3A expression was associated with a more advanced stage in NPC.

### Inhibition of TGF-β1 by SB431542 Increased T Cells Activity by Inhibiting PD-L1 Glycosylation *In Vitro*

**Figure 5:**
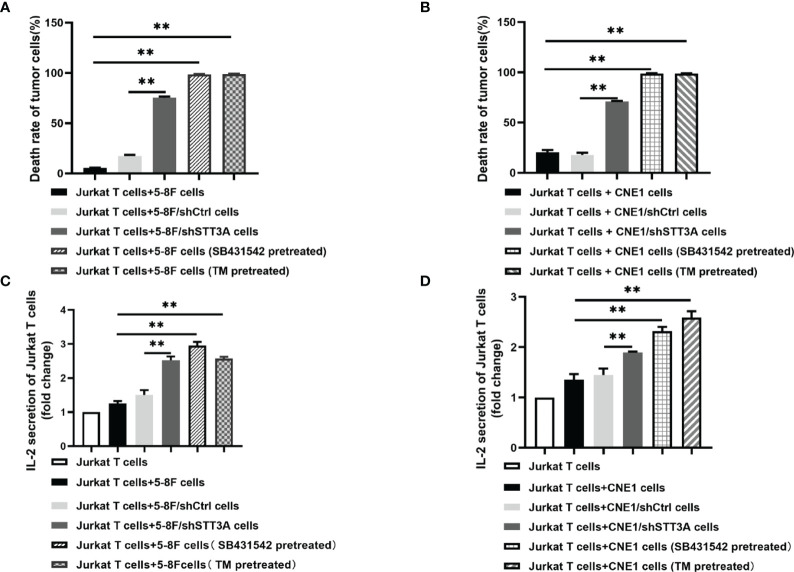
Inhibition of TGF-β1 by SB431542 suppressed T cells activity by inhibiting PD-L1 glycosylation in Vitro. **(A, B)** The death rate of 5-8F or CNE1 cells co-cultured with Jurkat T cells for 6h. The tumor cells were STT3A-kncokdowned by shRNA or pretreated with SB431542 (20μM) or TM (10μg/ml) for 48h. **(C, D)** IL-2 production by activated Jurkat T cells co-cultured with 5-8F or CNE1 cells with shSTT3A or pretreated with SB431542 (20μM) or TM (15μg/ml) for 48h. IL-2 secretion was measured by ELISA. Bars, mean ± SD, **p<0.01.

The above results have confirmed that TGF-β1 promoted PD-L1 glycosylation in NPC through c-Jun/STT3A pathway, and we wondered whether TGF-β1 induced immunosuppression by mediating PD-L1 glycosylation. A co-culture system containing NPC cells and Jurkat T cells was conducted to detect the effect of STT3A-knockdown, SB431542 and tunicamycin (TM) on T cells. The results showed that compared with control group, STT3A-knockdown group had greatly increased tumor cell death rate but lower than SB431542 or TM-treated groups (**Figures 5A, B**). We also detected IL-2 level in the supernatant of the co-culture system, and found that in SB431542 and TM pretreated groups, the secretion of IL-2 by T cells was highest, and IL-2 level of STT3A-knockdown group also had a significant increase compared with control group (**Figures 5C, D**). The above results suggested that immunosuppression in NPC induced by inhibition of TGF-β1 was largely dependent on STT3A-regulated PD-L1 glycosylation.

## Discussion

Immunotherapy is one of the most encouraging treatment for cancer patients, and the most common immunotherapy strategy includes the interruption of the interaction between immune checkpoints expressed on tumor and immune cells, especially targeting PD-L1/PD-1 (25). PD-L1 is an important immunosuppressive contributor to trigger immune escape by binding to its receptor PD-1 (26). Recent evidence has shown that the expression status of PD-L1, as detected by immunohistochemistry (IHC) has exhibited a significant correlation with response to immunotherapy, and patients whose tumors overexpress PD-L1 by IHC have improved clinical outcomes with anti-PD-1-directed therapy (27). Emerging studies have reported that NPC tumor cells highly express PD-L1, with a positive expression rate from 89% to 100% (28), however, only 20%-30% of NPC patients respond well to anti-PD-L1/PD-1 therapy (8–10). Therefore, understanding the regulation of PD-L1 expression are urgently needed to further improve response to immunotherapy.

Increasing studies have revealed that the expression and function of PD-L1 are regulated by PTMs (29). Among them, N-glycosylation is important in PD-L1/PD-1-mediated tumor immunosuppressive function and influences the efficacy of immunotherapy (30). Li CW et al. reported that glycosylation on N192/200/219 of PD-L1 inhibited the degradation of itself through suppressing GSK3β-β-Trcp-induced PD-L1 polyubiquitination (23). Moreover, PD-L1 glycosylation promoted its interaction with PD-1 and further suppressed T cells activity in triple-negative breast cancer (TNBC) (31). In addition, many studies suggested that targeting glycosylation of PD-L1 was an effective strategy to improve anti-tumor activity. In breast cancer, STT3 upregulated PD-L1 *via* increasing PD-L1 glycosylation in CSCs. Etoposide, which could inhibit EMT-mediated STT3 expression, enhanced the therapeutic outcomes of T cell immunoglobulin mucin-3 (TIM-3) blockade therapy (20). It was later reported that targeting PD-L1 glycosylation by 2-deoxy-D-glucose (2-DG) combined with EGFR inhibitors reduced tumor size and enhanced anti-tumor immunity mediated by 4-1BB, a glycoprotein receptor belonging to the tumor necrosis factor receptor superfamily, in syngeneic mouse models of TNBC (32). However, it remains unknown whether glycosylation of PD-L1 contributes to immune escape in NPC.

In our study, we confirmed that PD-L1 was heavily glycosylated in NPC, and found that TGF-β1 played an important role in N-glycosylation of PD-L1 by regulating glycotransferase STT3A in NPC. We also revealed that TGF-β1 could upregulate PD-L1 glycosylation and exert immunosuppressive effect *via* activating c-Jun/STT3A signaling pathway. Compared with the studies which reported that TGF-β1-induced EMT increased PD-L1 expression in NSCLC and TNBC, our data identified a novel pathway about TGF-β1-mediated PD-L1 glycosylation, further expanding the understanding of the regulatory mechanisms and cellular functions underlying PD-L1 glycosylation. Additionally, our data will also lead to the development of potentially effective therapeutics targeting PD-L1 glycosylation for clinical application.

As a driving factor of tumor progression, TGF-β can induce tumor cell plasticity by inhibiting the anti-tumor immune response, making epithelial tumor cells more mesenchymal, stem-like and resistant to immunotherapy. TGF-β also produces additional stromal modifiers that promote tumor progression and metastasis and shapes an immunosuppressive tumor microenvironment (16). Bintrafusp alfa is a dual-target inhibitor that can capture TGF-β while binding to PD-L1. This dual-target combination therapy can block the tumor-driving effect of TGF-β1 and overcome the immune escape induced by TGF-β1 (33), which provides more suitable conditions for the binding of monoclonal antibodies and PD-L1. It has been carried out in clinical trials of various tumors such as breast cancer, non-small cell lung cancer, prostate cancer, pancreatic cancer, bile duct cancer and cervical cancer and has achieved remarkable effects (34). In this study, we demonstrated that TGF-β1-mediated PD-L1 glycosylation promoted immune escape *via* c-Jun/STT3A pathway in NPC, which provided a theoretical basis for the clinical practice of bintrafusp alfa in NPC.

In summary, our study verified that TGF-β1 induced PD-L1 glycosylation and exerted immunosuppressive effect *via* c-Jun/STT3A signaling pathway in NPC. These results will provide a new understanding of the resistance mechanisms of anti-PD-1/PD-L1 therapy in NPC, and suggest that targeting TGF-β1 pathway might be a promising approach to enhance immune checkpoint blockade in NPC.

## Data Availability Statement

The original contributions presented in the study are included in the article/**Supplementary Material**. Further inquiries can be directed to the corresponding author.

## Ethics Statement

The studies involving human participants were reviewed and approved by Ethics Committee of Nanfang hospital. The patients/participants provided their written informed consent to participate in this study.

## Author Contributions

X-PL and JL conceived the study and drafted the manuscript. X-MM, Y-FL, FZ, and CS performed the experiments. XL contributed to the quality. All authors contributed to the article and approved the submitted version.

## Funding

This work was supported by National Natural Science Foundation of China (81472535) and Natural Science Foundation of Guangdong Province, China (2019A1515010968).

## Conflict of Interest

The authors declare that the research was conducted in the absence of any commercial or financial relationships that could be construed as a potential conflict of interest.

## Publisher’s Note

All claims expressed in this article are solely those of the authors and do not necessarily represent those of their affiliated organizations, or those of the publisher, the editors and the reviewers. Any product that may be evaluated in this article, or claim that may be made by its manufacturer, is not guaranteed or endorsed by the publisher.
