# Supplementary material for: TGF-β1-Mediated PD-L1 Glycosylation Contributes to Immune Escape via c-Jun/STT3A Pathway in Nasopharyngeal Carcinoma
Source: Front Oncol. 2022 Mar 4;12:815437. doi: 10.3389/fonc.2022.815437 (PMC8930841; doi:10.3389/fonc.2022.815437)
Supplement: Supplementary file 1 [file DataSheet_1.pdf]

Supplementary Table 1. Short-hairpin (shRNA) sequences used in this study

ID	5'	stem	loop	stem	3'
CD274-RNAi-a	Ccgg	ACCATCAAGTCCTGAGTGGTA	CTCGAG	TACCACTCAGGACTTGATGGT	TTTTTg
CD274-RNAi-b	aattcaaaaa	ACCATCAAGTCCTGAGTGGTA	CTCGAG	TACCACTCAGGACTTGATGGT	
JUN-RNAi-a	Ccgg	AAGATGGAAACGACCTTCTAT	CTCGAG	ATAGAAGGTCGTTTCCATCTT	TTTTTg
JUN-RNAi-b	aattcaaaaa	AAGATGGAAACGACCTTCTAT	CTCGAG	ATAGAAGGTCGTTTCCATCTT	
STT3A-RNAi-a	Ccgg	cgCAGCAAGTTGAATCCACAA	CTCGAG	TTGTGGATTCAACTTGCTGCG	TTTTTg
STT3A-RNAi-b	aattcaaaaa	cgCAGCAAGTTGAATCCACAA	CTCGAG	TTGTGGATTCAACTTGCTGCG	
