# Supplementary material for: TGF-β1-Mediated PD-L1 Glycosylation Contributes to Immune Escape via c-Jun/STT3A Pathway in Nasopharyngeal Carcinoma
Source: Front Oncol. 2022 Mar 4;12:815437. doi: 10.3389/fonc.2022.815437 (PMC8930841; doi:10.3389/fonc.2022.815437)
Supplement: Supplementary file 2 [file DataSheet_2.pdf]

Supplementary Table 2. All primers used in this study

A. qPCR primers

ID	Sequences
PD-L1 F	5’GGACAAGCAGTGACCATCAAG3’
PD-L1 R	5’CCCAGAATTACCAAGTGAGTCCT3’
c-Jun F	5’TCCAAGTGCCGAAAAAGGAAG3’
c-Jun R	5’ CGAGTTCTGAGCTTTCAAGGT3’
B3GNT3 F	5’CAGGACATGTGTGAGGCAAAAG3’
B3GNT3 R	5’TGAAGGGTATCTGAGCAGGGG3’
PGM3 F	5’GCAGAGAGTGCTTATTGACATCA3’
PGM3 R	5’TGTGAAAGTTTCTCACTGCTGG3’
STT3A F	5’CAAAGAGCACGAGATGAAA3’
STT3A R	5’TCTGGAATGCCTGAAATAG3’
STT3B F	5’AGTAGGTGGTACTGTTTACCCAG3’
STT3B R	5’AAGTTGGTGCAAGGAACACAC3’
TMEM165 F	5’TTGTCGCTGCCATATCAGTTATT3’
TMEM165 R	5’GGCGGTTATAGCGCATTGC3’
VCP F	5’CAAACAGAAGAACCGTCCCAA3’
VCP R	5’TCACCTCGGAACAACTGCAAT3’
MOGS F	5’AGTGACTGTAGAGCCTCAGGA3’
MOGS R	5’CCCACTGATAAACTTCAACTGCC3’
ALG8 F	5’AGTACACCGAAACTGGCTTGC3’
ALG8 R	5’GTAATCCAACGTCCACTCTGAAG3’
DPAGT1 F	5’TGGCAACACGACCATTGTG3’
DPAGT1 R	5’CCCCATGTAGACATAGTACAGGA3’
DDOST F	5’GAGACTCATTCGCTTTTCTTCCG3’
DDOST R	5’CTCCAAAATCTTCTACCGAAGGG3’
RPN1 F	5’ACTCTCCCTATCCAACGAAGAC3’
RPN1 R	5’CCCAGCTTGGTGTAGCTC3’
RPN2 F	5’ACAACAGCGTTATTTGTGGCT3’
RPN2 R	5’TCGGAGAGGGACTCAAAGTTC3’
GAPDH F	5’TGTGGGCATCAATGGATTTGG3’
GAPDH R	5’ACACCATGTATTCCGGGTCAAT3’

B. CHIP-PCR primers

ID	Sequences
CHIP 1F	5’TCCCACTCTCTCTGGTGTAGC3’
CHIP 1R	5’TATCCTAACTTGCCTCTCTTC3’
CHIP NCF	5’GGAGATCGAGGCCATCCTGG3’
CHIP NCR	5’TGTAGGTTATACAACTTTGGC3’

C. Dual-luciferase assay primers

ID	Sequences
mutSTT3A 1F	5'AACAAAGGGCTGACTGACTGCCATATGGCCGGGCTG

	CCTCCCGGTCTCCCGAGGAA3'
mutSTT3A 1R	5'GCCCGGCCATATGGCAGTCAGTCAGCCCTTTGTTAAA ATACCGGCCAGAGAGCCGC3'
mutSTT3A 2F	5'TCTTTCCGCGCTCTGACTGTTGCTATGCCGGCCTATG ACAGCTCGGCGGGAGCATA 3'
mutSTT3A 2R	5'TAGGCCGGCATAGCAACAGTCAGAGCGCGGAAAGA ACGTGCCCCAAGCAGTTTGGT 3'
c-Jun F	5'ATGACTGCAAAGATGGAAACGACC 3'
c-Jun R	5'TCAAAATGTTTGCAACTGCTGC 3'
STT3A F	5'GCTCTTGCTGCCCAGACTGAAGTGCAATGG 3'
STT3A R	5'GGCAGGAGCCGGGTCCGCCTCCCGGACCGC 3'
