# Supplementary material for: TGF-β1-Mediated PD-L1 Glycosylation Contributes to Immune Escape via c-Jun/STT3A Pathway in Nasopharyngeal Carcinoma
Source: Front Oncol. 2022 Mar 4;12:815437. doi: 10.3389/fonc.2022.815437 (PMC8930841; doi:10.3389/fonc.2022.815437)
Supplement: Supplementary file 3 [file DataSheet_3.pdf]

Supplementary Table 3. Clinical characteristics of the 36 patients with NPC

Characteristic	N(%)
Sex	
Male	10 (27.8%)
Female	26 (72.2%)
Age (years)	
<55	29 (80.6%)
≥55	7 (19.4%)
Clinical stage[1]	
I	-
II	6 (16.7%)
III	16 (44.4%)
IVa	11 (30.6%)
IVb	3 (8.3%)
Tumor stage[1]	
T1	4 (11.1%)
T2	5 (13.9%)
T3	19 (52.8%)
T4	8 (22.2%)
Node stage[1]	
N0	1 (2.8%)
N1	10 (27.8%)
N2	11 (30.6%)
N3	14 (38.9%)
Metastasis stage[1]	
M0	34 (94.4%)
M1	2 (5.6%)

NPC, nasopharyngeal carcinoma

[1]According to the 8th edition of the UICC/AJCC staging system.
