# Supplementary material for: TGF-β1-Mediated PD-L1 Glycosylation Contributes to Immune Escape via c-Jun/STT3A Pathway in Nasopharyngeal Carcinoma
Source: Front Oncol. 2022 Mar 4;12:815437. doi: 10.3389/fonc.2022.815437 (PMC8930841; doi:10.3389/fonc.2022.815437)
Supplement: Supplementary file 4 [file DataSheet_4.docx]

Supplementary Material

# 1.Supplementary Figures
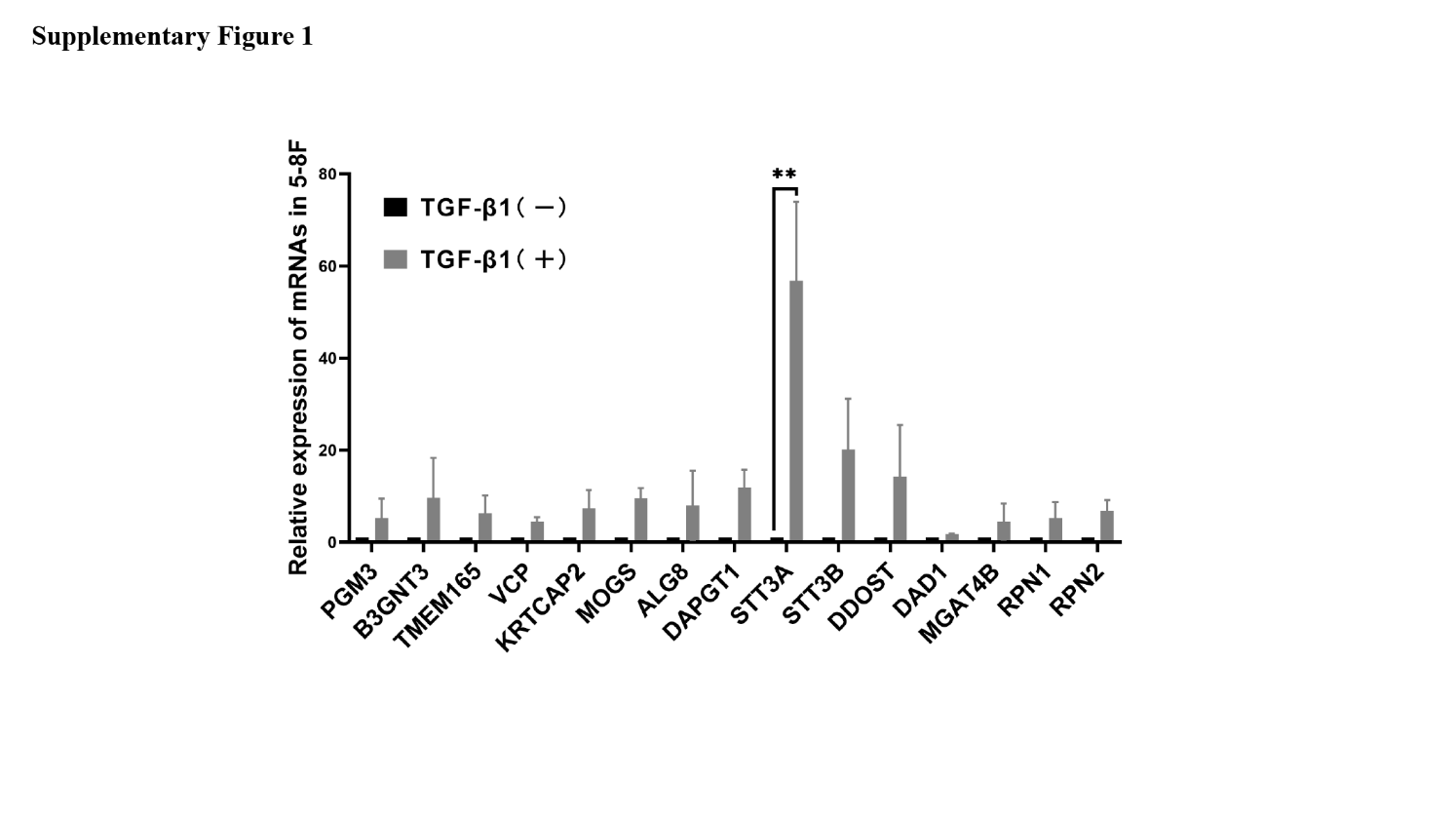


**Supplementary Figure 1.** The mRNA levels of 15 glycosyltransferases related to PD-L1 glycosylation in 5-8F cell after the treatment of TGF-β1 by RT-qPCR.

**
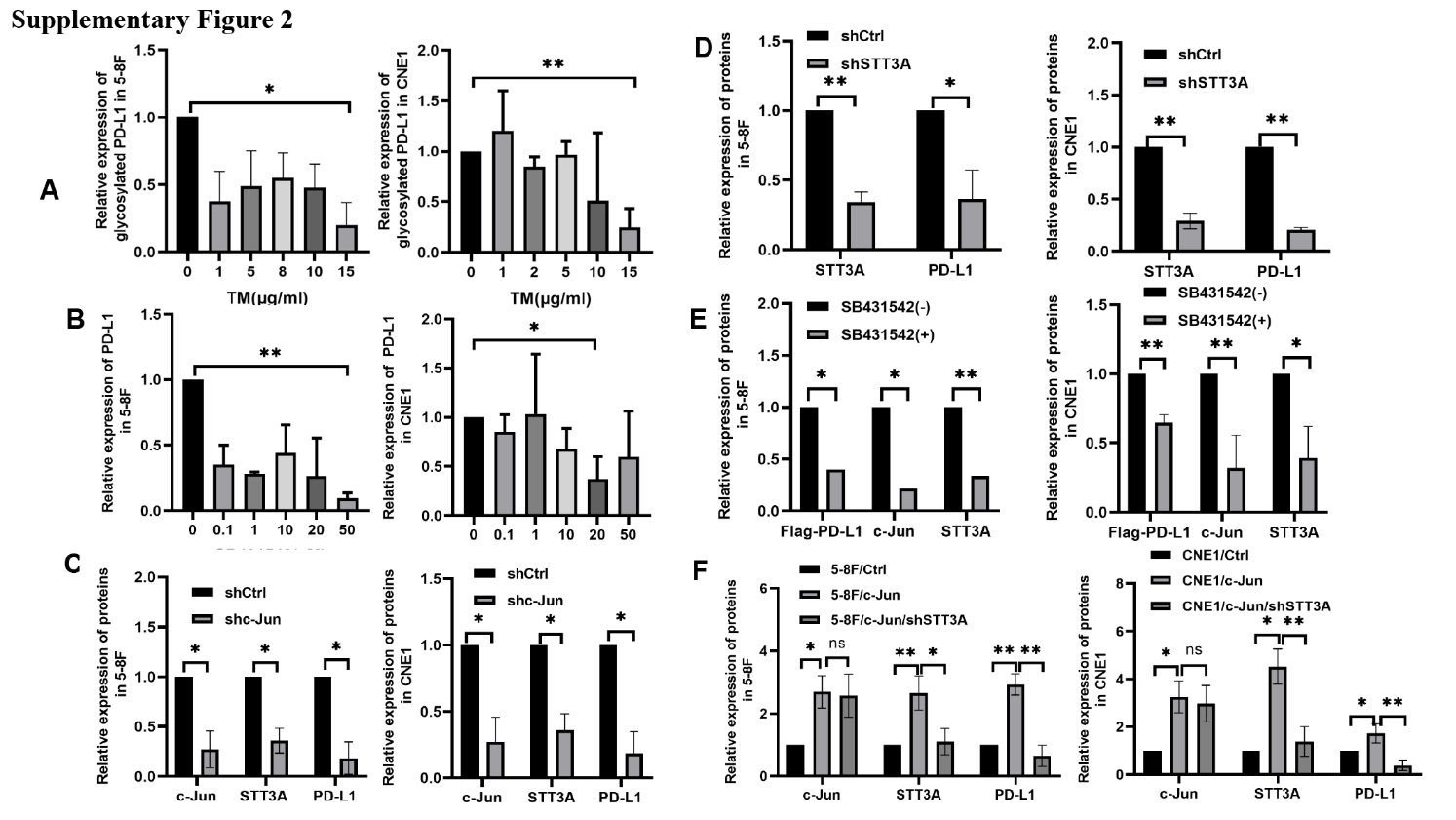
**

**Supplementary Figure 2. Statistical analysis of Western Blotting.(A)** The glycosylated PD-L1 was inhibited by tunicamycin (TM) at a concentration of 15 μg/ml in 5-8F and CNE1. **(B)** SB431542 suppressed the expression of PD-L1 in 5-8F and CNE1. **(C)** After silencing the expression of c-Jun with shRNA, STT3A and PD-L1 were downregulated. **(D)** With the knockdown of STT3A, the expression of PD-L1 was downregulated in 5-8F and CNE1. **(E)** SB431542 suppressed the expression of Flag-PD-L1, c-Jun and STT3A in Flag-PD-L1-5-8F and Flag-PD-L1-CNE1. **(F)** Overexpression of c-Jun upregulated the expression of STT3A and PD-L1, and the upregulation was reversed by the knockdown of STT3A in 5-8F and CNE1. Bars means± SD, **p*<0.5, ***p*<0.01,

# 2. Supplementary Tables

**Supplementary Table 1.** Short-hairpin (shRNA) RNA sequences used in this study.

**Supplementary Table 2.** All primers used in this study.

**Supplementary Table 3.** Clinical characteristics of the 36 patients with NPC.

(Supplementary tables were shown in the additional files.)
